# Supplementary figures and images for: Mec1-independent activation of the Rad53 checkpoint kinase revealed by quantitative analysis of protein localization dynamics
Source: eLife. 2023 Jun 6;12:e82483. doi: 10.7554/eLife.82483 (PMC10259420; doi:10.7554/eLife.82483)

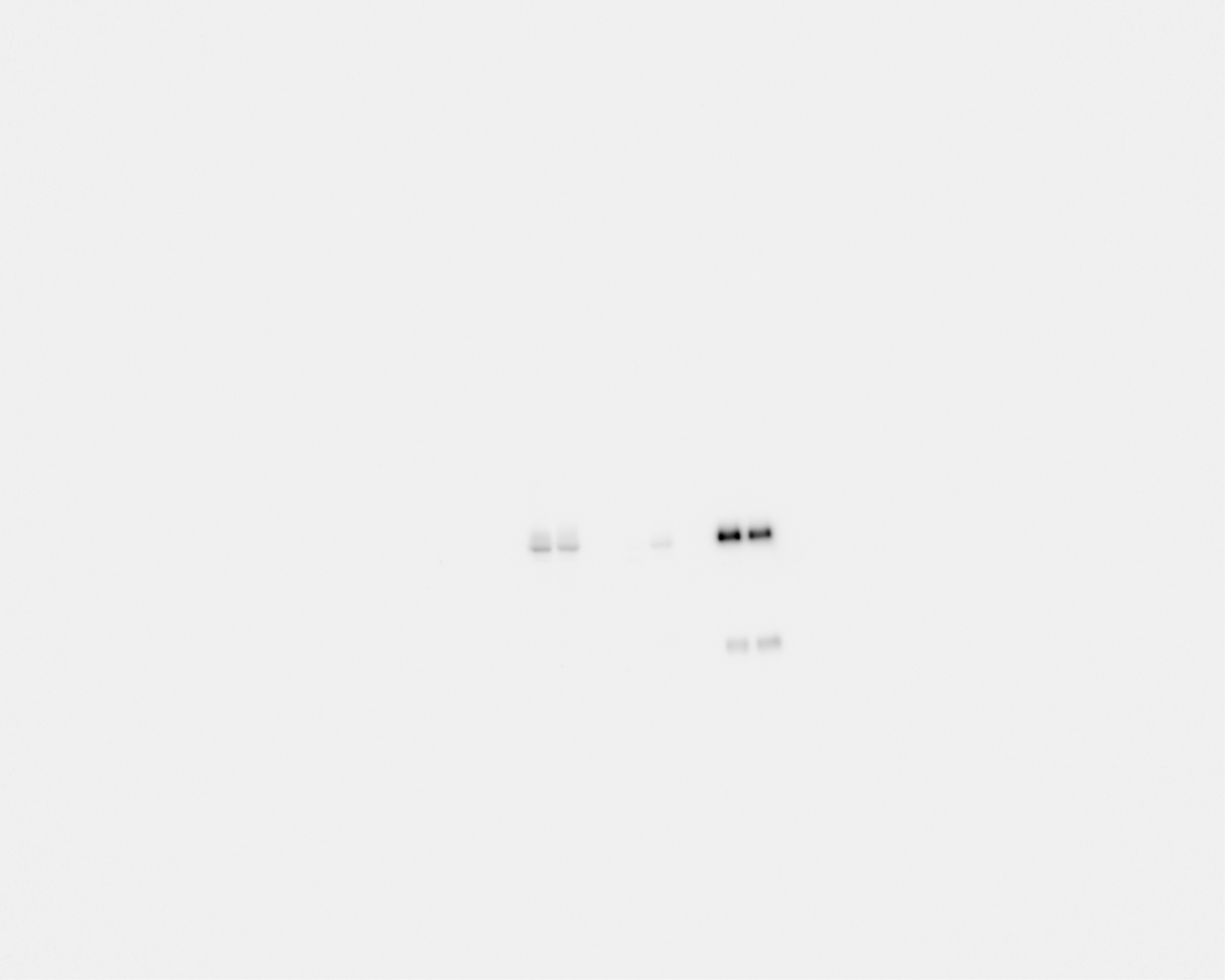

Supplement: Figure 6—figure supplement 2—source data 1. [file elife-82483-fig6-figsupp2-data1.zip › Figure 6-Supplement 2-Source Data 1/Figure 6-Supplement 2-Raw Uncropped Immunoblots for Rad53-FLAG Immunoprecipitation Full Image.png]
