# Supplementary material for: Mec1-independent activation of the Rad53 checkpoint kinase revealed by quantitative analysis of protein localization dynamics
Source: eLife. 2023 Jun 6;12:e82483. doi: 10.7554/eLife.82483 (PMC10259420; doi:10.7554/eLife.82483)
Supplement: MDAR checklist [file elife-82483-mdarchecklist1.pdf]

eLife

Westbrook Centre
Milton Road
Cambridge CB4 1YG, UK

P 01223 855340
W elifesciences.org
T @elife

Materials Design Analysis Reporting (MDAR) Checklist for Authors

The MDAR framework establishes a minimum set of requirements in transparent reporting mainly applicable to studies in the life sciences.

eLife asks authors to **provide detailed information within their article** to facilitate the interpretation and replication of their work. Authors can also upload supporting materials to comply with relevant reporting guidelines for health-related research (see EQUATOR Network), life science research (see the BioSharing Information Resource), or animal research (see the ARRIVE Guidelines and the STRANGE Framework; for details, see *eLife*'s Journal Policies). Where applicable, authors should refer to any relevant reporting standards materials in this form.

For all that apply, please note **where in the article** the information is provided. Please note that we also collect information about data availability and ethics in the submission form.

Materials:

Newly created materials	Indicate where provided: section/figure legend	N/A
The manuscript includes a dedicated "materials availability statement" providing transparent disclosure about availability of newly created materials including details on how materials can be accessed and describing any restrictions on access.	Materials and Methods Section, Computational Analyses Data, and Software Availability subheading (Page 17): links to code and statement to image availability provided.	

Antibodies	Indicate where provided: section/figure legend	N/A
For commercial reagents, provide supplier name, catalogue number and RRID, if available.	Materials and Methods Section, Whole cell extracts and immunoblotting subheading (Page 16): Rad53 (1:5000, Abcam ab104232), anti-PGK (1:1 000 000, Novex 459250), anti-GFP (1:5000, ClonTech 632375), and anti-FLAG M2 (1:5000, Sigma F3165)	

DNA and RNA sequences	Indicate where provided: section/figure legend	N/A
Short novel DNA or RNA including primers, probes: Sequences should be included or deposited in a public repository.		No novel DNA or RNA sequences used

**Cell materials**	**Indicate where provided: section/figure legend**	**N/A**
Cell lines: Provide species information, strain. Provide accession number in repository OR supplier name, catalog number, clone number, OR RRID.		No cell lines used in present study
Primary cultures: Provide species, strain, sex of origin, genetic modification status.		No primary cultures used in present study

**Experimental animals**	**Indicate where provided: section/figure legend**	**N/A**
Laboratory animals or Model organisms: Provide species, strain, sex, age, genetic modification status. Provide accession number in repository OR supplier name, catalog number, clone number, OR RRID.		No animals used in present study
Animal observed in or captured from the field: Provide species, sex, and age where possible.		No animals used in present study

**Plants and microbes**	**Indicate where provided: section/figure legend**	**N/A**
Plants: provide species and strain, ecotype and cultivar where relevant, unique accession number if available, and source (including location for collected wild specimens).		No plants used in present study
Microbes: provide species and strain, unique accession number if available, and source.	Materials and Methods Section, Yeast strains subheading (Page 13): *Saccharomyces cerevisiae* used in this study (S288C BY4741 background)	

**Human research participants**	**Indicate where provided: section/figure legend) or state if these demographics were not collected**	**N/A**
If collected and within the bounds of privacy constraints report on age, sex, gender and ethnicity for all study participants.		No human participants used in present study

Design:

**Study protocol**	**Indicate where provided: section/figure legend**	**N/A**
If the study protocol has been pre-registered, provide DOI. For clinical trials, provide the trial registration number OR cite DOI.		This study is not registered for clinical trials.

**Laboratory protocol**	**Indicate where provided: section/figure legend**	**N/A**
Provide DOI OR other citation details if detailed step-by-step protocols are available.		Detailed step-by-step protocols are not available.

**Experimental study design (statistics details) ***		
**For in vivo studies: State whether and how the following have been done**	**Indicate where provided: section/figure legend. If it could have been done, but was not, write “not done”**	**N/A**
Sample size determination		In vivo studies not present in our study.
Randomisation		In vivo studies not present in our study.
Blinding		In vivo studies not present in our study.
Inclusion/exclusion criteria		In vivo studies not present in our study.

**Sample definition and in-laboratory replication**	**Indicate where provided: section/figure legend**	**N/A**

State number of times the experiment was replicated in the laboratory.	High-throughput confocal microscopy screens were not replicated; Rad54-GFP kinase-associated screen was performed three independent times.	
Define whether data describe technical or biological replicates.	Figure captions with indicated replicates and/or multiple independent measurements/observations (Fig 1b, 2, 3, 4c, 5c) are biological replicates.	

**Ethics**	**Indicate where provided: section/submission form**	**N/A**
Studies involving human participants: State details of authority granting ethics approval (IRB or equivalent committee(s), provide reference number for approval.		No human participant were used in this study.
Studies involving experimental animals: State details of authority granting ethics approval (IRB or equivalent committee(s), provide reference number for approval.		No experimental animals were used in this study.
Studies involving specimen and field samples: State if relevant permits obtained, provide details of authority approving study; if none were required, explain why.		No field samples or specimens were used in this study.

**Dual Use Research of Concern (DURC)**	**Indicate where provided: section/submission form**	**N/A**
If study is subject to dual use research of concern regulations, state the authority granting approval and reference number for the regulatory approval.		Not applicable; data collected in this study used for this study alone.

Analysis:

**Attrition**	**Indicate where provided: section/figure legend**	**N/A**

Describe whether exclusion criteria were pre-established. Report if sample or data points were omitted from analysis. If yes, report if this was due to attrition or intentional exclusion and provide justification.		No exclusion criteria, and data points were not omitted from any analyses performed in this study.

**Statistics**	**Indicate where provided: section/figure legend**	**N/A**
Describe statistical tests used and justify choice of tests.	Materials and Methods Section: Data analysis with SEQUEST software and estimation of False Discovery Rates (FDR) for SILAC-MS. These parameters are established and commonly used in literature to identify changes in phosphopeptides by MS.<br><br>Fig 2g and 4b: Two sided T-test statistical test between replicates.<br><br>Materials and Methods Section, Calculating percentages of cells exhibiting a localization subheading: algorithms and statistical tests used in initial analysis that have been established in previous preprint/study.	

**Data availability**	**Indicate where provided: section/submission form**	**N/A**
For newly created and reused datasets, the manuscript includes a data availability statement that provides details for access (or notes restrictions on access).	Materials and Methods Section, Computational Analyses Data, and Software Availability subheading (Page 17): links to code and statement to image availability provided.	
When newly created datasets are publicly available, provide accession number in repository OR DOI and licensing details where available.	Materials and Methods Section, Computational Analyses Data, and Software Availability subheading (Page 17): datasets are provided in supplemental tables, and images are currently in submission at IDR microscopy open access.	

If reused data is publicly available provide accession number in repository OR DOI, OR URL, OR citation.		Reused data is not used in our study.

Code availability	Indicate where provided: section/figure legend	N/A
For any computer code/software/mathematical algorithms essential for replicating the main findings of the study, whether newly generated or re-used, the manuscript includes a data availability statement that provides details for access or notes restrictions.	Materials and Methods Section, Computational Analyses Data, and Software Availability subheading (Page 17): links to code and statement to image availability provided. GitHub link is provided, no restrictions, open access, the repository is made public.	
Where newly generated code is publicly available, provide accession number in repository, OR DOI OR URL and licensing details where available. State any restrictions on code availability or accessibility.	GitHub link is provided, no restrictions, open access, the repository is made public. This information is in the Materials and Methods Section.	
If reused code is publicly available provide accession number in repository OR DOI OR URL, OR citation.		No publicly available and reused code was used in our study.

Reporting:

The MDAR framework recommends adoption of discipline-specific guidelines, established and endorsed through community initiatives.

Adherence to community standards	Indicate where provided: section/figure legend	N/A
State if relevant guidelines (e.g., ICMJE, MIBBI, ARRIVE, STRANGE) have been followed, and whether a checklist (e.g., CONSORT, PRISMA, ARRIVE) is provided with the manuscript.		No additional guidelines or checklists are provided with the manuscript.

---

* We provide the following guidance regarding transparent reporting and statistics; we also refer authors to Ten common statistical mistakes to watch out for when writing or reviewing a manuscript.

Sample-size estimation

- You should state whether an appropriate sample size was computed when the study was being designed

- You should state the statistical method of sample size computation and any required assumptions
- If no explicit power analysis was used, you should describe how you decided what sample (replicate) size (number) to use

Replicates

- You should report how often each experiment was performed
- You should include a definition of biological versus technical replication
- The data obtained should be provided and sufficient information should be provided to indicate the number of independent biological and/or technical replicates
- If you encountered any outliers, you should describe how these were handled
- Criteria for exclusion/inclusion of data should be clearly stated
- High-throughput sequence data should be uploaded before submission, with a private link for reviewers provided (these are available from both GEO and ArrayExpress)

Statistical reporting

- Statistical analysis methods should be described and justified
- Raw data should be presented in figures whenever informative to do so (typically when N per group is less than 10)
- For each experiment, you should identify the statistical tests used, exact values of N, definitions of center, methods of multiple test correction, and dispersion and precision measures (e.g., mean, median, SD, SEM, confidence intervals; and, for the major substantive results, a measure of effect size (e.g., Pearson's r, Cohen's d)
- Report exact p-values wherever possible alongside the summary statistics and 95% confidence intervals. These should be reported for all key questions and not only when the p-value is less than 0.05.

Group allocation

- Indicate how samples were allocated into experimental groups (in the case of clinical studies, please specify allocation to treatment method); if randomization was used, please also state if restricted randomization was applied
- Indicate if masking was used during group allocation, data collection and/or data analysis
